# Cytoglobin as a Marker of Hepatic Stellate Cell-derived Myofibroblasts

**DOI:** 10.3389/fphys.2015.00329

**Published:** 2015-11-13

**Authors:** Norifumi Kawada

**Affiliations:** Department of Hepatology, Graduate School of Medicine, Osaka City UniversityOsaka, Japan

**Keywords:** stellate cell, cytoglobin, myofibroblast, liver fibrosis, hypoxia

## Abstract

Myofibroblasts play important roles in inflammation, fibrosis and tumorigenesis in chronically inflamed liver. Liver myofibroblasts originate from hepatic stellate cells, portal fibroblasts or mesothelial cells, and they are localized in and around fibrotic septum and portal tracts. Liver myofibroblasts are the source of extracellular matrix materials, including type I collagen and multiple fibrogenic growth factors, such as transforming growth factor-β and vascular endothelial growth factor. Although a detailed characterization of the function of individual myofibroblasts has not been conducted, owing to the lack of appropriate cell markers, recent lineage-tracing technology has revealed the limited contribution of myofibroblasts that are derived from portal fibroblasts to various types of liver fibrosis, as compared with the contribution of hepatic stellate cells. In addition, cytoglobin, which is the fourth globin in mammals and function as a local gas sensor, provides a new perspective on the involvement of stellate cells in fibrosis and carcinogenesis, possibly through its anti-oxidative properties and is a promising new marker that discriminates between myofibroblasts derived from stellate cells and those from portal fibroblasts.

## Introduction

The myofibroblast was originally described by Gabbiani in the granulation tissue of healing wounds as a cell type that had characteristics of fibroblasts and smooth muscle cells (Gabbiani et al., 1971).

In the liver, myofibroblasts were first identified in hepatic schistosomal fibrosis (Grimaud and Borojevic, 1977); this was followed by the identification of contractile fibroblasts (myofibroblasts) in chronic alcoholic cirrhosis (Rudolph et al., 1979).

Liver myofibroblasts are featured by the expression of α-smooth muscle actin (α-SMA), which is encoded by *ACTA2* gene located in 10q22-q24 in the human genome (Schmitt-Gräff et al., 1991). The origin of liver myofibroblasts is complex: at least three cellular sources—hepatic stellate cells (HSCs), portal fibroblasts (PFs), and mesothelial cells (MCs)—are considered to transdifferentiate to liver myofibroblasts (Yin et al., 2013). The contribution of each type of myofibroblast to the development or resolution of liver fibrosis varies depending on the etiology of liver disease.

Conversely, cytoglobin (Cygb) belongs to the mammalian globin family, which consists of myoglobin, hemoglobin, neuroglobin, and androglobin (Burmester and Hankeln, 2014). Cygb was accidentally discovered in rat HSCs during the proteomics analysis of proteins that are expressed in HSCs. The amino acid sequence and gas-binding ability of Cygb are similar to those of myoglobin, thus suggesting that these two globins have the same primary function in pathophysiology (Kawada et al., 2001; Burmester et al., 2002; Trent and Hargrove, 2002). However, recent studies have suggested that Cygb is involved in the carcinogenesis of various organs, which is different from the function of myoglobin, a gas-binding sensor that affects muscle cell contraction (Shivapurkar et al., 2008; Shaw et al., 2009; Latina et al., 2015).

In this review, we will discuss the role of Cygb, which is uniquely expressed in HSCs in the liver, in the myofibroblastic transformation of HSCs and carcinogenesis in the liver.

## Liver myofibroblasts

In 2002, Cassiman et al. proposed the presence of at least four different subpopulations of myofibroblasts in human liver. Portal myofibroblasts and septal myofibroblasts are located in the extended collagenous area around portal tracts and in the internal region of fibrotic septa, respectively. These myofibroblasts are strongly positive for α-SMA, and they exhibit a variable positivity to glial fibrillar acidic protein (GFAP), brain-derived nerve growth factor (BDNF) and α-B-crystallin (ABCRYS). The third type of myofibroblasts is interface myofibroblasts, which present at the edge between fibrotic septa and the surrounding hepatic parenchyma, which are positive for α-SMA, GFAP, and ABCRYS as well as BDNF, neuronal cell adhesion molecule (N-CAM), neuronal growth factor (NGF) and neurotrophin 4 (NT-4). The fourth type is activated HSCs that are located in or around sinusoidal capillary and within pseudo-lobules and express intensively α-SMA and N-CAM and are positive for GFAP, NGF, BDNF, NT-3, and synaptophysin as well as NT-4, ABCRYS, p75 and the NT receptors tyrosine kinase A and B. Cassiman et al. proposed that portal myofibroblasts, in addition to activated HSCs, may originate from PFs (Cassiman et al., 2002). Recent cell-tracing technology has clarified the origin of myofibroblasts in further detail, as discussed below.

### Hepatic stellate cells

HSCs reside in Disse's space in the hepatic sinusoid, a space between sinusoidal endothelial cells and hepatocytes (Wake, 1980). HSCs constitute approximately 10% of total liver cells in the adult healthy liver. In healthy situation, quiescent HSCs principally store vitamin A in droplets in their cytoplasm. It is calculated that HSCs in the liver store 45–72% of vitamin A in the body. Quiescent HSCs are positive for neural crest markers like GFAP and NTs, nerve growth factor receptor (p75), and desmin. Quiescent HSCs are the source of extracellular matrix materials (ECMs) and the component of basement membrane-like structure, such as laminin, proteoglycan, and type IV collagen (Friedman, 2008; Yin et al., 2013; Pellicoro et al., 2014). HSCs also function as liver-specific pericytes surrounding sinusoidal endothelial cells, and thus their contractility is regulated by the exposure to endothelin-1 (ET-1), angiotensin-II, and their relaxation by nitric oxide (NO) control the diameter of sinusoids and regulates the hepatic microcirculation (Kawada et al., 1993).

Liver trauma caused by hepatitis virus B or C infection, alcohol abuse, drug toxicity, autoimmunity, or steatohepatitis, triggers the activation of HSCs and their transdifferentiation from quiescent phenotype to myofibroblast-like one (Novo et al., 2014). The myofibroblast-like HSCs contain less amount of vitamin A droplets, express increased α–SMA and growth factor receptors, possess augmented contractility, and generate multiple ECMs, including type I and III collagens (Friedman, 2008; Yin et al., 2013; Pellicoro et al., 2014). This activation process of HSCs is initiated by paracrine stimulation by neighboring and activated sinusoidal endothelial cells, Kupffer cells (liver macrophages), hepatocytes and cholangiocytes, as well as platelets. When activated, HSCs synthesize transforming growth factor β (TGF-β), which stimulates HSCs themselves as an autocrine loop. Hepatic sinusoidal endothelial cells take part in the activation of TGF-β from its latency-associated peptide-binding form by its proteolytic cleavage (Schuppan and Kim, 2013). Cholangiocytes and platelets are also an important origin of growth factors, including platelet-derived growth factor (PDGF), TGF-β, and epidermal growth factor. Activation of HSCs is additionally initiated by damaged hepatocytes; they secrete fibrogenic lipid peroxides, and their apoptosis mediated by Fas and tumor-necrosis factor (TNF)-related apoptosis-inducing ligand initiates the activation of HSCs via damage-associated molecular pattern molecules including high-mobility group box 1, RNA and DNA, S100 molecules, and purine metabolites. In culture system, it was also demonstrated that apoptotic fragments derived from damaged hepatocytes stimulate HSC activation, and that fibrogenic activity of myofibroblasts are augmented by the phagocytosis of apoptotic hepatocytes due to NADPH oxidase 2 and the janus kinase/signal transducer and activator of transcription and phosphoinositide 3-kinase/Akt pathways (Wree et al., 2014). Activated HSCs also produce an increased amount of matrix metalloproteinases (MMPs), especially MMP13, and their inhibitors, tissue inhibitor of matrix metalloproteinases (TIMPs).

Transcription factors including activated protein-1, Jun D, Sp1, Kruppel-like factor 6, and nuclear factor kappa B, whose functions are strictly regulated by intracellular signaling molecules, such as Smad, Ras, Raf-1, and mitogen-activated protein kinase, control the activation of HSC, leading to the transcriptional upregulation of latent TGF-β (Mann and Smart, 2002; Lopez-Sanchez et al., 2014). Epigenetic regulation, specifically DNA methylation and histone modification, is also important in the regulation of the activation of HSCs. For example, the abnormal DNA methylation of phosphatase and tension homolog (PTEN) and MeCP2 has been identified in activated HSCs (Lee et al., 2015). Among epigenetic signals, microRNAs also participate in the activation of HSCs. The microRNAs that have been implicated in HSC activation include miR-199a,b, miR-221, miR-27, miR-21, miR-125, miR-195, miR-214, and miR-221/222 as profibrotic miRNAs, and miR-29, miR-15b, miR-200, miR-16, miR-133b, and miR-122 as anti-fibrotic miRNAs. Among these miRNAs, miR-29 has been the most intensively analyzed for its ability to suppress collagen 1A1 production in HSCs (Sekiya et al., 2011; Ogawa et al., 2012). In addittion, the augmented production of TIMPs impedes ECM degradation and triggers ECM accumulation in the damaged liver (Perugorria et al., 2013). It should be noted that leptin and other adipocytokines are involved in the process of HSC activation (Choi et al., 2010) and that activated HSCs are additionally characterized by augmented expression of receptors for PDGF, TGF-β, vascular endothelial growth factor (VEGF), angiotensin-II, and ET-1 (Novo et al., 2012). Through study of reporter Collagen α1(I)-GFP (Col-GFP) mice, GFP^+^Vitamin-A^+^Desmin^+^ activated HSCs have been shown to comprise more than 92% of myofibroblasts in the fibrotic liver induced by CCl_4_ intoxication (Scholten et al., 2010).

Hepatocellular carcinoma (HCC) is a consequence of chronic liver disease with many etiologies, and it develops after repetitive hepatocyte death and the development of fibrosis. In fact, it is thought that 80% of HCC develop in fibrotic and cirrhotic liver. Thus, fibrosis consisting of HSC-derived myofibroblasts may be involved in cancer development. Because activated HSCs are the source for growth factors and ECMs as mentioned above, they may contribute to the development of a microenvironment that is suitable for cancer growth. In fact, Coulouarn et al. demonstrated the importance of HSC-hepatocyte interaction due to the production of VEGFA and MMP-9 (Coulouarn et al., 2012). Very recently, Yoshimoto et al. elucidated that senescent HSCs activated by a bile acid derived from intestinal microbiota shows a senescent-associated secretary phenotype, in which a panel of cytokines, such as IL-1, IL-6, and GROα, are synthesized. This phenomenon is suggested in the central scenario for the development of liver cancer in obesity (Yoshimoto et al., 2013).

### Portal fibroblasts

PFs are the resident fibroblasts around the portal tract existing in the mesenchyme surrounding the bile ducts. PFs were first described more than 50 years ago in rat liver. It is considered that PFs in addition to HSC-derived myofibroblasts produce ECMs particularly in case of biliary fibrosis (Wells, 2014). Although it is considered that the septum tranversum-derived mesothelial cells are origin of PFs and HSCs, the definition of the roles of PFs in normal and injured liver has been unsatisfactory confirmed due to the lack of reliable markers to distinguish PFs from HSCs. PFs are generally positive for gremlin, Thy-1, fibulin 2, elastin, interleukin 6 (IL-6), cofilin, and the ectonucleotidase NTPDase 2. Lemoinne et al. demonstrated that COL15A1-positive portal and periportal cells give rise to portal myofibroblasts and release VEGFA-laden microparticles, thus promoting endothelial tubulogenesis (Lemoinne et al., 2015). Unlike HSCs, PFs lack desmin, cytoglobin, β2-macroglobulin, Hand2, GFAP, p75^NGFr^, and Vitamin A. Using a mouse model carrying a bacterial artificial chromosome with a Cre reporter derived from the lecithin-retinol acyltransferase, Mederacke et al. recently showed that HSCs are the source of 82–96% of myofibroblasts in seven models of fibrosis in mice, although they could not eliminate the possibility that PFs are required for biliary fibrosis. Particularly, bridging fibrosis occurs from the portal vein area to the central vein area where PFs and desmin–negative cells are localized (Mederacke et al., 2013). Iwaisako et al. showed the important role of myofibroblasts derived from PFs during the initial stages of bile-duct ligation-induced liver fibrosis by contributing more than 70% of myofibroblasts, although HSC-derived myofibroblasts become dominant at the last phase (Iwaisako et al., 2014). In their study, Iwaisako et al. also identified mesothelin as a useful marker to distinguish PF-myofibroblasts from HSC-myofibroblasts.

PFs can be differentiated from HSCs-myofibroblasts by their production of elastin. As mentioned before, PFs were originally defined by their fibulin 2 expression (Knittel et al., 1999). Fibulin 2 is a linker protein that occupies the interface between microfibrils and their elastin core. Fibulin 2 mediates the progression of fibrosis by sequestering latent TGF-β binding proteins from TGF-β. In addition, PFs produce lysyl oxidase (LOX) and the related protein LOX-like 1, which cross-links elastin. Taken together, the function of PFs should be estimated regarding to the metabolism of elastin since elastin fibers are able to provide the bile ducts and the portal vasculature with mechanical stability (Perepelyuk et al., 2013).

The interaction of PFs with cholangiocytes is another important issue. Tanimizu et al. described that neurotrophin receptor p75-positive progenitors of PFs and cholangiocytes express laminin α1 and α5 subunits, respectively, which interact with the β1 integrin subunit to maintain the polarity and lumen of the bile ducts (Tanimizu et al., 2012). On the other hand, Jhandier et al. reported that PF-derived myofibroblasts do not express ectonucleotidase NTPDase2, which is equivalent to ecto-ATPase or CD39L1 and metabolizes extracellular nucleotides. This fact may explain how cholangiocyte proliferation is initiated by P2Y receptor activation in chronic cholangiopathies (Jhandier et al., 2005).

### Mesodermal mesenchymal cells

Although myofibroblasts are able to be derived from epithelial cells like hepatocytes and cholangiocytes, during the epithelial-to-mesenchymal transition, recent genetic cell lineage tracing has clearly revealed that mesenchymal cells including HSCs, PFs, and vascular smooth muscle cells are derived from MCs in the mouse embryonic liver. In addition, in adult Wt1^CreERT2^; R26T/G^f^ mice after the induction of fibrosis by CCl_4_ or bile duct ligation, MCs that are positive for glycoprotein M6a, podoplanin and CD200 acquire mesenchymal characteristics—expression of collagen 1α1 and vimentin—but the epithelial cell markers are decreased. This phenomenon of the conversion of liver MCs to mesenchymal cells is called the mesothelial-mesenchymal transition. The canonical TGF-β pathway plays a central role in MMT via SMAD3 (Li et al., 2013; Yin et al., 2013; Lua et al., 2014).

## Cytoglobin

Cygb belongs to the mammalian globin family including myoglobin in muscle cells, hemoglobin in red blood cells, neuroglobin in the nervous system, and androglobin in testis. Cygb, originally named stellate cell activation-associated protein by our group and histoglobin by Trent and Hagrove, was discovered in 2001 from cultured rat HSCs by proteomics analysis, namely the separation of proteins expressed in HSCs by 2D SDS-PAGE, followed by the identification of individual proteins by time of flight mass spectrometry. The molecular weight of Cygb is 21,496 Da, and it is composed of 190 amino acids (Kawada et al., 2001; Trent and Hargrove, 2002; Burmester and Hankeln, 2014).

**Figure 1:**
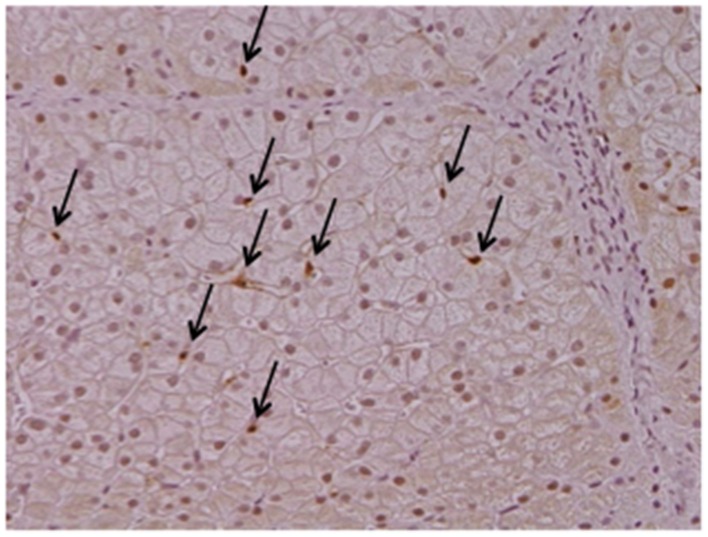
**Cytoglobin expression in intact human liver**. Cytoglobin (arrows) is localized along hepatic sinusoid, namely these cells are identical to hepatic stellate cells, but not hepatocytes. Intact human liver tissue was obtained at Osaka City University Hospital under the patient's consent (Motoyama et al., 2014). Written informed consent was obtained from the patient. NK contributed to the evaluation of diagnosis of human liver histopathology.

Cygb is uniquely expressed in HSCs, but not in hepatocytes, endothelial cells, or Kupffer cells, in the liver (Figure 1). Cygb is also expressed in extrahepatic organs, including the pancreas, spleen, kidney, digestive tract, and heart. Cygb-positive cells usually localize proximally to capillaries, and they are considered to be pericytes that store vitamin A, produce collagen and closely correlate with vascular endothelium (Nakatani et al., 2004). In addition, Cygb is expressed in chondrocytes, osteoblasts and osteocytes (Schmidt et al., 2004). Furthermore, a recent report has indicated that Cygb is expressed in melanocytes and several types of melanoma, and it is involved in the melanocyte-to-melanoma transition through hyper-methylation in the promoter region of the CYGB gene (Fujita et al., 2014).

The 3D structure of Cygb is almost identical to that of myoglobin, and it has a three-over-three α-helical sandwich structure consisting of eight helices, whereas Cygb is a bis-histidyl hexa-coordinate globin similar to neuroglobin and different from myoglobin and hemoglobin, which are penta-coordinate globins. However, Cygb can bind gas molecules including oxygen, nitric oxide, and carbon monoxide with kinetic properties similar to those of myoglobin. Thus, Cygb is thought to function as a local gas-sensor (Makino et al., 2006, 2011). A recent report has indicated the role of Cygb as a nitric oxide dioxygenase [Cygb(Fe^2+^) → O_2_ → Cygb(Fe^2+^−O_2_) → NO → Cygb(Fe^3+^) + ${\text{NO}}_{3}^{-}$] resulting in the regulation of the local concentration of nitric oxide and hence vascular tonus (Liu et al., 2012).

Cygb gene expression is regulated by hypoxia inducible factor-1 (HIF-1), which binds to the hypoxia response element of the Cygb gene promoter. In fact, exposure of HeLa cells to 1% O_2_ upregulates Cygb mRNA expression 1.7-fold (3 h) and 1.6-fold (6 h) in culture. HIF-1 and erythropoietin have 2 (-141 and -448) and 1 (-144) binding site, respectively, within the 5′-UTR lesion of the Cygb gene; these sites are important for Cygb gene transcription under hypoxia (Guo et al., 2007). The other transcription factors involved in Cygb gene expression include c-Ets-1, Sp1, nuclear factor of activated T cell and activator protein (Guo et al., 2006). In case of the liver, Cygb is induced during hypoxia (24 and 48 h at 7% O2) and reoxygenetion in mice (Fordel et al., 2007). Man et al. demonstrated that more than 3.5-fold upregulation of Cygb mRNA expression and the increase in the number of Cygb-expressing cells after the administration of CCl_4_ in mice (Man et al., 2008). Gninsky et al. reported the induction of Cygb in thioacetamide-intoxicated liver fibrosis model in rats, which was clearly suppressed by the treatment with halofuginone, an inhibitor of TGF-β-dependent Smad3 phosphorylation (Gnainsky et al., 2007). In culture studies, Cui et al. demonstrated the induction of Cygb in primary-cultured rat HSCs by arundic acid, an inhibitor of S100b synthesis in astrocytes (Cui et al., 2012). Stone et al. recently clarified the regulation of Cygb expression in HTC-T6 cells in culture; Cygb expression is upregulated by laminin via integrin α1β4 signaling while it is downregulated by type I collagen by phosphorylation of focal-adhesion kinase via integrin α2β1 signaling (Stone et al., 2015).

Although Cygb's function as a fas-binding molecule is well-established, its pathophysiological role has not been fully elucidated. Cygb protects SH-SY5Y neuroblastoma cells from apoptosis under exposure to 300 μM H_2_O_2_ (Fordel et al., 2006). Li et al. demonstrated that siCygb treatment facilitates N2a neuronal cell death under exposure to 500 μM H_2_O_2_ for 4 h (Li et al., 2007). These and other studies have revealed that Cygb is a possible cytoprotector against oxidative stress.

Recently, the role of Cygb in human carcinogenesis in various organs has been paid special attention. The downregulation of Cygb gene expression has been demonstrated in human esophageal cancer, head and neck cancer, lung cancer, ovarian cancer and prostatic cancer via the hyper-methylation of the promoter lesion of the Cygb gene (Shivapurkar et al., 2008; Shaw et al., 2009; Latina et al., 2015). In detail, Oleksiewicz et al. showed (1) marked down-regulation of Cygb protein in human lung cancer cell line compared to normal human epithelial cell lines and possible involvement of promoter methylation and (2) significantly reduced Cygb mRNA expression in non-small cell lung cancer tissues compared with normal lung tissue (Mann-Whitney test, *P* = 2.3 × 10^−7^), suggesting the possible tumor suppressor function of Cygb (Oleksiewicz et al., 2013). Based on these reports, Hubers et al. studied Cygb gene methylation in spontaneous sputum in order to utilize it for lung cancer diagnosis. As a result, they reported the sensitivity of Cygb for the diagnosis of lung cancer was 22% (Hubers et al., 2014). Except for the lung cancer, Wojnarowicz et al. demonstrated that Cygb gene expression was lowered in the majority of tumors with low malignant potential and cancer compared to benign tumors and normal ovarian surface epithelial cell samples (Wojnarowicz et al., 2012). Chen et al. also clarified the involvement of Cygb in ovarian cancer development at human tissue level and in cell culture models (Chen et al., 2014). Similar observations were additionally reported in human atrophy and adenocarcinoma of the prostate, suggesting that prostatic malignancy is accompanied with low level of Cygb, which play a role in protecting from oxidative damage (Mogal et al., 2012).

Using Cygb-knockout mice that were generated in our laboratory, we studied the role of Cygb in liver cancer development. First, mice were treated with 0.05 ppm diethylnitrosamine, an established carcinogen to the liver, for 36 weeks, thus resulting in the occurrence of liver tumors in 57.1% of the knockout mice compared with 0% of the wild-type mice. In this model, background liver tissues showed a marked development of liver fibrosis, augmented inflammatory reactions and overproduction of peroxynitrite (ONOO^−^) in knockout mice (Thuy le et al., 2011). Second, mice were given a choline-deficient L-amino acid-defined diet for 32 weeks to induce steatohepatitis. Unexpectedly, 100% of Cygb knockout mice developed multiple liver tumors compared with 0% of the wild type mice. Again, background liver tissues showed marked development of liver fibrosis and augmented inflammatory reactions, which were accompanied by DNA double strand breaks (γH2AX expression) in hepatocytes. These results show the protective role of Cygb against oxidative stress and liver fibrosis development under chronic inflammation, suggesting Cygb's role in tissue carcinogenesis (Thuy le et al., 2015). In accordance with these observations, previous two reports demonstrated the anti-fibrotic function of Cygb. Xu et al. reported that Cygb overexpression in the liver by recombinant adeno-associated virus-2 encoding full-length rat Cygb suppressed liver injury and fibrosis induced by CCl_4_ adminstration or bile-duct ligation in rats (Xu et al., 2006) and, furthermore, He at al. showed that administration of recombinant Cygb (10 mg/Kg) attenuates liver fibrosis in thioacetamide-induced liver fibrosis in rats (He et al., 2011), suggesting the hepato-protective role of Cygb.

## Cytoglobin as a marker of hepatic stellate cell-derived myofibroblasts

**Figure 2:**
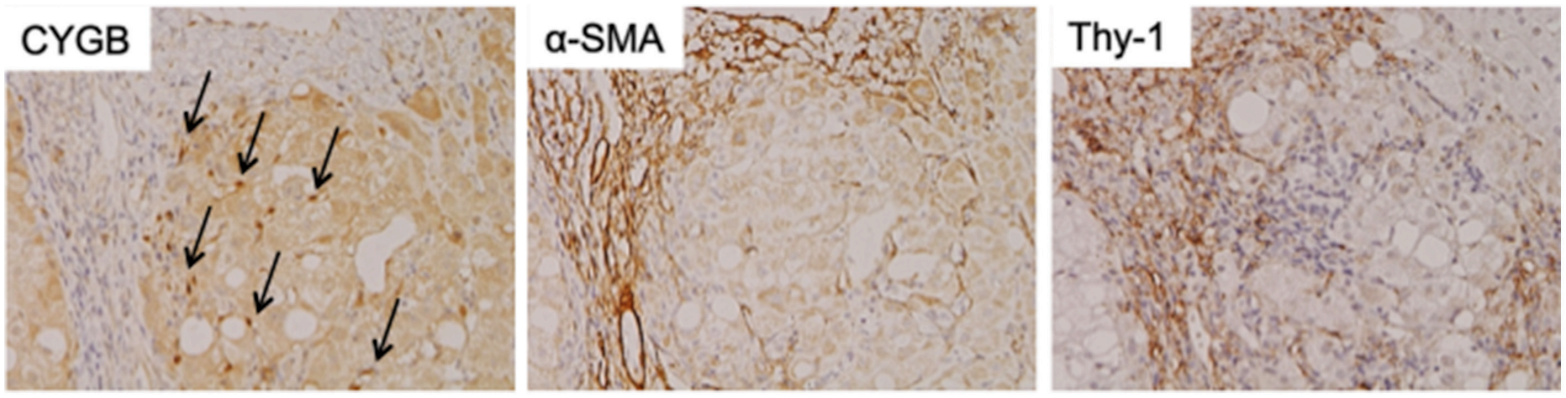
**Cytoglobin expression in fibrotic human liver**. Cytoglobin (arrows) is localized along hepatic sinusoid, while α-SMA and Thy-1, myofibroblast markers, were strongly positive at fibrotic septum. Human liver tissue with fibrosis caused by chronic hepatitis C was obtained at Osaka City University Hospital under the patient's consent (Motoyama et al., 2014). Written informed consent was obtained from the patients. NK contributed to the evaluation of diagnosis of human liver histopathology.

As mentioned above, Cygb was initially discovered from rat HSCs in primary culture (Kawada et al., 2001). Cygb is expressed in mouse and human HSCs (Figure 1). Thus, whether Cygb can be utilized as a specific marker to distinguish myofibroblasts derived from HSCs from those derived from PFs is an interesting issue. Schmidt et al. first provided a detailed description of Cygb-expressing cells *in vivo* in mice, and they demonstrated that, in the liver, Cygb is expressed in HSCs and fibroblasts that surround the portal vein, but not in hepatocytes (Schmidt et al., 2004). However, in this study, fibroblasts around the portal vein were insufficiently characterized. Using fibulin 2 as a PF marker, Tateaki et al. demonstrated that there are several types of fibroblastic cells in the liver, namely fibulin 2^+^/Cygb^+^, fibulin 2^+^/Cygb^−^, fibulin 2^−^/Cygb^+^, fibulin 2^−^/Cygb^−^, and they concluded that fibulin 2^+^/Cygb^+^ and fibulin 2^−^/Cygb^+^ are identical to portal myofibroblasts and activated HSCs, respectively. A limitation of this study is that the identification of individual cell types was performed only by immunofluorescence staining (Tateaki et al., 2004). Ogawa et al. isolated the mouse liver non-parenchymal cell fraction by the Nycodenz density gradient method and further separated the cells in the fraction on the basis of vitamin A autofluorescence with FACS. UV^+^ cells had minimum growth potential, expressed desmin and Cygb, but not αSMA and fibulin 2 at 1 day in culture, and later became positive for desmin, Cygb, and αSMA at 7 day in culture. Hence, UV^+^ cells (vitamin A-storing cells) are identical to HSCs. Conversely, UV^−^ cells showed high proliferation activity and expressed αSMA, fibulin 2, and Gremlin, but not desmin and Cygb, thus indicating that UV^−^ cells are derived from portal fibroblasts (Ogawa et al., 2007). Bosselut at al. demonstrated that isolated rat portal myofibroblasts do not express Cygb, and thus are different from rat HSCs, as determined by 2-D MS/MS (Bosselut et al., 2010). Recent study by Fausther et al. described that rat portal fibroblasts and portal myofibroblast cell lines, RGF and RGF-N2, express Cygb at least in mRNA level in culture model (Fausther et al., 2015). In contrast, Motoyama et al. demonstrated that in human liver damaged by chronic hepatitis C, Cygb- and cellular retinol-binding protein-positive cells localize along hepatic sinusoids in liver parenchyma and at the margin of fibrotic septum, and they are not identical to αSMA, fibulin 2, and Thy-1-positive myofibroblasts (Motoyama et al., 2014; Figure 2). In summary, in mouse, rat, and human liver, Cygb is expressed in quiescent HSCs, and its expression level increases in activated HSCs that also express αSMA. In contrast, myofibroblasts derived from PFs are positive for fibulin 2 and αSMA, and they are negative for Cygb in human, while in rodent Cygb expression in PF-derived myofibroblasts needs to be characterized further by, for example, using genetic cell-labeling technology.

## Author contributions

NK contributed to the evaluation of diagnosis of human liver histopathology.

### Conflict of interest statement

The author declares that the research was conducted in the absence of any commercial or financial relationships that could be construed as a potential conflict of interest.

## Abbreviations

ABCRYS: α-B-crystallin
α-SMA: α-smooth muscle actin
BDNF: brain-derived nerve growth factor
Cygb: cytoglobin
ET-1: endothelin-1
ECM: extracellular matrix material
GFAP: glial fibrillar acidic protein
HSC: hepatic stellate cell
HIF: hypoxia inducible factor
LOX: lysyl oxidase
MMP: matrix metalloproteinase
MC: mesothelial cell
NGF: neuronal growth factor
NT: neurotrophin
NO: nitric oxide
PDGF: platelet-derived growth factor
PF: portal fibroblast
TIMP: tissue inhibitor of matrix metalloproteinases
TGF-β: transforming growth factor β
TNF: tumor-necrosis factor
VEGF: vascular endothelial growth factor.
